# Minocycline ameliorates cognitive impairment induced by whole-brain irradiation: an animal study

**DOI:** 10.1186/s13014-014-0281-8

**Published:** 2014-12-12

**Authors:** Liyuan Zhang, Kun Li, Rui Sun, Yuan Zhang, JianFeng Ji, Peigeng Huang, Hongying Yang, Ye Tian

**Affiliations:** Department of Radiotherapy and Oncology, Second Affiliated Hospital, Soochow University, 1055 Sanxiang Road, Suzhou, Jiangsu Province 215004 PR China; Jiangsu Key Laboratory of Translational Research and Therapy for Neuro-Psycho-Diseases, Second Affiliated Hospital, Soochow University, 1055 Sanxiang Road, Suzhou, Jiangsu Province 215004 PR China; Collaborative Innovation Center of Radiation Medicine of Jiangsu Higher Education Institutions, Suzhou, Jiangsu Province 215123 PR China; Department of Medical Oncology, Affiliated Hospital of Taishan Medical University, Taian, Shandong Province 271000 PR China; Department of Radiation Oncology, Massachusetts General Hospital, Harvard Medical School, 100 Blossom Street, Boston, MA 02114 USA; School of Radiation Medicine and Protection, Medical College of Soochow University/School for Radiological and Interdisciplinary Sciences (RAD-X), Soochow University, 199 Renai Road, Suzhou Industrial Park, Suzhou, Jiangsu Province 215123 PR China

**Keywords:** Whole-brain irradiation, Cognitive deficit, Minocycline, Newborn neuron, Apoptosis, Neurogenesis

## Abstract

**Background:**

It has been long recognized that cranial irradiation used for the treatment of primary and metastatic brain tumor often causes neurological side-effects such as intellectual impairment, memory loss and dementia, especially in children patients. Our previous study has demonstrated that whole-brain irradiation (WBI) can cause cognitive decline in rats. Minocycline is an antibiotic that has shown neuroprotective properties in a variety of experimental models of neurological diseases. However, whether minocycline can ameliorate cognitive impairment induced by ionizing radiation (IR) has not been tested. Thus this study aimed to demonstrate the potential implication of minocycline in the treatment of WBI-induced cognitive deficits by using a rat model.

**Methods:**

Sprague Dawley rats were cranial irradiated with electron beams delivered by a linear accelerator with a single dose of 20 Gy. Minocycline was administered via oral gavages directly into the stomach before and after irradiation. The open field test was used to assess the anxiety level of rats. The Morris water maze (MWM) was used to assess the spatial learning and memory of rats. The level of apoptosis in hippocampal neurons was measured using immunohistochemistry for caspase-3 and relative markers for mature neurons (NeuN) or for newborn neurons (Doublecortin (DCX)). Neurogenesis was determined by BrdU incorporation method.

**Results:**

Neither WBI nor minocycline affected the locomotor activity and anxiety level of rats. However, compared with the sham-irradiated controls, WBI caused a significant loss of learning and memory manifest as longer latency to reach the hidden platform in the MWM task. Minocycline intervention significantly improved the memory retention of irradiated rats. Although minocycline did not rescue neurogenesis deficit caused by WBI 2 months post-IR, it did significantly decreased WBI-induced apoptosis in the DCX positive neurons, thereby resulting in less newborn neuron depletion 12 h after irradiation.

**Conclusions:**

Minocycline significantly inhibits WBI-induced neuron apoptosis, leading to less newborn neurons loss shortly after irradiation. In the long run, minocycline improves the cognitive performance of rats post WBI. The results indicate a potential clinical implication of minocycline as an effective adjunct in radiotherapy for brain tumor patients.

## Background

As an important treatment modality for primary and metastatic brain tumors, cranial irradiation often causes neurological side-effects such as intellectual impairment, memory loss and dementia, especially in children patients [1-4]. The cognitive decline has been suggested to be due to radiation-induced deficits in the hippocampal-dependent functions of learning, memory and spatial information processing [5-8]. Although the mechanisms underlying radiation-induced cognitive impairment remain to be elucidated, the studies using radiation-induced learning and memory deficit animal models have shown that the decline of hippocampal-dependent functions is generally accompanied by hippocampal apoptosis [8,9], decreased hippocampal proliferation [9,10], reduced neurogenesis [11,12], and marked alteration in neurogenic microenvironment [7,13].

In attempt to alleviate the neurotoxicity of radiotherapy for brain tumor patients and improve the quality of their life after treatment, intense effort is being made to develop the methods that can attenuate radiation-induced cognitive impairment. For example, exercise [14,15], transplantation of human fetal-derived neural stem cells [16], and some pharmacological agents such as Lithium compound [8,17,18] have been shown to improve cognitive function post irradiation.

Minocycline, a clinical available antibiotic, has been demonstrated to be neuro-protective in animal models of several acute central neural system (CNS) injuries and neurodegenerative diseases [19,20]. In the present study, we tested whether minocycline could inhibit radiation-induced cognitive decline. We found that minocycline intervention significantly attenuated the learning and memory loss caused by whole-brain irradiation (WBI) 2 months post-irradiation. Our short-term study showed that minocycline significantly prevented hippocampal neurons, especially DCX+ neurons from WBI-induced apoptosis 6 hours post-irradiation, thus leading to less newborn neurons loss. However, minocycline had no effect on neurogenesis deficit 2 months after WBI. The results indicate a potential implication for minocycline in ameliorating radiation-induced cognitive dysfunction.

## Methods

### Animals and experimental groups

All animal procedures were carried out in accordance with Soochow University Medical Experimental Animal Care Guidelines based on the National Animal Ethical Policies. One-month-old male Sprague Dawley rats weighing 90-110 g (obtained from the Experimental Animal Center of Soochow University) were used as described previously [21]. The animals were housed in cages at an ambient temperature of 22 ± 1°C with a 12-h light-dark cycle. Pelleted rat chow and tap water were available ad libitum.

Rats were randomly allocated into six groups: untreated control (CN), minocycline (CM), sham control (SCN), sham minocycline (SCM), radiation (RN) and minocycline plus radiation (RM) (n = 18/group except the SCN and SCM groups which had n = 12/group). The CN group or the CM group received only saline or minocycline, respectively; The SCN group or the SCM group were subjected to the radiation procedure with 0 Gy in addition to receiving saline or minocycline. The CN, CM, SCN and SCM groups are referred as the control groups in the text. The RN group or the RM group were subjected to WBI in addition to the treatment with either saline or minocycline.

### Minocycline treatment

One day before radiation, rats received either a total dose of 90 mg/kg of clinical grade minocycline (100 mg/capsule, Huishi Pharmaceutical Ltd. Co., P. R. China) dissolved in saline in 2 divided doses or the same volume of physiological saline alone (vehicle, 4.5 ml/kg) via oral gavages directly into the stomach. After irradiation, animals were administered with either saline or minocycline twice daily (45 mg/kg/d) for 2 months [19].

### Irradiation

Prior to irradiation, animals were anesthetized with 3.6% chloral hydrate (360 mg/kg, i.p.). Then the WBI was performed using 4-MeV electron beams delivered by a linear accelerator (Philips SL − 18, Philips, UK) at room temperature (RT), as described previously [22]. Briefly, a 20 × 20 cm lead shielding block with 10 holes specifically cut for WBI was used. The size of each hole was ~ 3.5 × 2.0 cm (length × width). One hole was for one rat brain. The other parts of rat’s body were shielded with the lead block. For irradiated rats, a single WBI dose of 20 Gy was given at a dose rate of 210-220 cGy/min.

### General observation and body weight gain

After irradiation, rats were monitored for their motor activity, feeding and drinking behavior, as well as the side effects such as nausea, ataxia, and topical skin reaction. Their body weights were recorded biweekly. All these observations were recorded and used as parameters of general changes after treatment.

### Open field test

Since the level of anxiety could affect the performance of learning and memory test, open field test was used to assess the anxiety level of all groups as described previously [21]. Briefly, three days after 2-month minocycline intervention, open field test was performed in a silent dimly lit room. The open field used in this study, which was a square sound proof chamber (410 × 410 × 505 mm), was made of Plexiglas so that rats were visible from outside of the chamber. The floor of the chamber was divided into 25 8 × 8 cm squares, and the area that contained 9 squares in the middle was called the central region. A video camera was placed above the center of the open field. The rats were placed in the central region and allowed to move freely and to explore the environment for 10 min. The movements were recorded by computer-aided video-tracking system (Jiliang, Shanghai, China). The total distance rats traveled around the open field area and the time they spent in the central region of the open field were analyzed. After each individual test, the apparatus was cleaned with 10% ethanol thoroughly to remove any olfactory cues.

### Morris water maze (MWM)

The MWM is generally used to assess the spatial learning and memory of rats [23]. The experimental apparatus, which is a circular water tank (160 cm diameter, Jiliang, Shanghai, China) filled with opaque water (22 ± 1°C), and has a hidden submerged platform (9 cm diameter) in the center of the target quadrant [23], was used to assess the ability of a rat to locate the platform. The rats were placed into each of the 4 quadrants of the pool for 60 s, and were trained to locate the submerged hidden platform. They had to find the platform using only the distal spatial cues available in the testing room. When failed, the rats were gently placed on the platform for 10 s. The time rats spent on searching and mounting the platform (i.e. latency) and their swim speeds were recorded by video-tracking system (Jiliang, Shanghai, China). After 4 days of place navigation tests, a 30-s spatial probe test (the submerged platform removed) was performed on day 5. The time rats spent on crossing the target quadrant and all four quadrants were recorded for 30 s by the tracking system.

### Assessment of neurogenesis by BrdU incorporation

Four weeks after sham or WBI treatment, the rats were injected intraperitoneally with a dose of BrdU (50 mg/kg, Sigma, St Louis, MO, USA) daily for 6 consecutive days. Three weeks after the last dose of BrdU, rats were sacrificed and the brain tissues were harvested and processed using the method below for analysis of neurogenesis.

### Tissue preparation and immunohistochemistry

Rats were sacrificed at 3, 6 or 12 h after irradiation for apoptosis measurement or 2 months post-irradiation for neurogenesis studies. To remove the brains, anesthetized rats were transcardially perfused with PBS followed by decapitation, then the brains were placed in 10% paraformaldehyde solution for 24 h. A single 5-mm-thick section containing the hippocampus was dissected and paraffin-embedded, and the brain levels were approximately 125 ± 150 μm apart as previously described [9]. At least ten non-overlapping coronal sections (4 μm) were cut from three different brain levels by using a RM 2135 microtome (Leica, Germany) and mounted on poly-L-lysine-coated slides.

The tissue sections were processed as previously described [9] and double stained with mouse monoclonal anti-NeuN (specific to human, mouse, rat and chicken NeuN, 1:100, Millipore, USA) or goat anti-DCX antibodies (specific to mouse, rat and human DCX, 1:50, Santa Cruz, USA) and rabbit polyclonal anti-active caspase-3 antibodies (specific to mouse, rat, human and quail active caspase-3, 1:15, Abcam, UK). After incubation with primary antibodies, the sections were washed and incubated sequentially with their respective secondary antibodies for 1.5 hours at RT, which are Alexa Fluor® 594 donkey anti-mouse secondary antibodies (1:100, Invitrogen, USA) for NeuN or Cy3 donkey anti-goat secondary antibodies (1:250, Beyotime, China) for DCX and Alexa Fluor® 488 donkey anti-rabbit secondary antibodies (1:150, Invitrogen, USA) for activated caspase-3. Then the sections were washed in PBS, counter stained with 4′,6′-diamidimo-2-phenylindole (DAPI, Sigma-Aldrich, USA) and sealed with nail polish. The number of NeuN/active caspase-3 double positive cells in the granule cell layer (GCL) and DCX/active caspase-3 double positive cells within the subgranular zone (SGZ) of the suprapyrimidal and infrapyrimidal blades of the dentate gyrus were scored blindly using a histomorphometric approach [9] under a fluorescent microscope (DM 2000, Leica, Germany).

For analysis of neurogenesis in the SGZ, the sections were dual-immunostained with NeuN and BrdU (1:120, Abcam, UK) antibodies. Then the sections were washed and sequentially incubated with the secondary antibody for NeuN and Alexa Fluor® 488 donkey anti-rat secondary antibody (1:200, Invitrogen, USA) for BrdU for 1.5 hours at RT. After washing, the sections were also stained with DAPI. Cell counts were limited to the GCL and a 50-μm border along the hilar margin that included the SGZ. And all samples were scored blindly. For each animal, five to six sections from three regions of the hippocampus were analyzed. The total number of positively labeled cells was determined by adding up the numbers of positive cells in both dentate gyri from all analyzed sections in the same rat. And all immunofluorescent images were captured using a Nikon confocal fluorescent microscope (A1, Japan).

### Statistics

The results were expressed as mean ± SEM. The cognitive study data were analyzed via one-way ANOVA followed by a Tukey post hoc test for multiple comparison using OriginPro Software (v8.0). And the immunohistochemical study data were analyzed using two-sample *t*-test. A *P* < 0.05 between groups was considered significantly different.

## Results

### General observation and body weight gain

**Figure 1:**
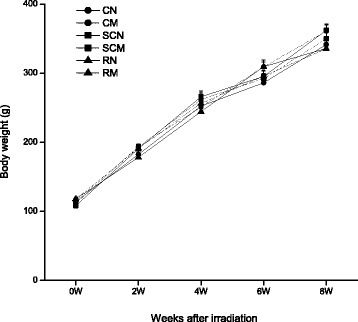
**Body weight gain in all groups of rats within two months after WBI.** There was no significant difference in body weights among the six groups (*P* > 0.05). The number of rats: n = 18/group except the SCN and SCM groups which had n = 12/group.

All rats receiving WBI survived for at least two months, and showed normal motor activities and feeding and drinking behavior. A few irradiated rats showed mild local skin reactions and depilation at the irradiated spot from 2 to 6 weeks after WBI, but all irradiated rats did not have radiation sickness symptoms such as nausea, ataxia or edema. Moreover, all groups showed steady body weight gain within two months after radiation. There was no statistically significant difference in the body weight among the six groups during the observation period (*P* > 0.05, Figure 1), suggesting that anesthesia, minocycline and radiation did not affect the growth of rats.

### Minocycline ameliorated radiation-induced cognition impairment

**Figure 2:**
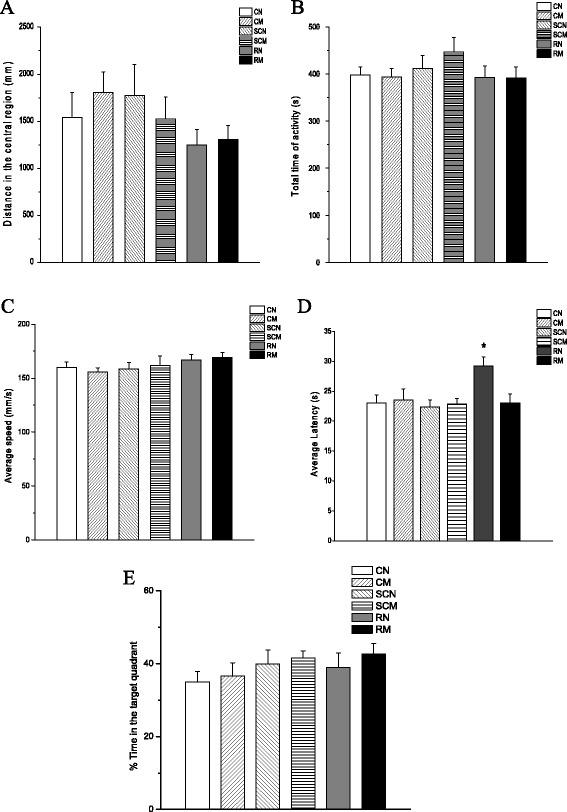
**Behavioral test. (A)** The distance all groups of rats traveled in the central region was determined in the open field test. No significant difference was observed (*P* > 0.05). **(B)** The six groups did not show any statistical difference in the total time of activity in the open field test (*P* > 0.05). **(C)** The six groups showed similar swimming speeds in the Morris water maze test (*P* > 0.05). **(D)** A single dose of 20 Gy WBI significantly increased latency, e.g. the time they needed to reach the hidden platform in the MWM test (*P <* 0.05 vs. unirradiated control). And minocycline decreased the latency back to the control level (*P* > 0.05 vs. unirradiated controls, but *P <* 0.05 vs. irradiated group). **(E)** No significant difference in the percentage of the target quadrant exploring time among the six groups (*P* > 0.05). The number of rats: n = 18/group except the SCN and SCM groups which had n = 12/group.

The open field test shows that there was no significant difference in the distance rats traveled in the central region and the total time of activity among six groups (*P* > 0.05) (Figure 2A, B). This indicated that anesthesia, WBI and minocycline had no effect on the locomotor activity and the anxiety level of rats. In MWM test, no significant difference in swimming speeds among these six groups was observed (*P* > 0.05) (Figure 2C). In the place navigation test, compared with the SCN group, rats in the RN group showed longer latency, e.g. the time they needed to reach the hidden platform, after irradiation (*P* = 0.027). Minocycline alone did not have any effect on the latency (*P* > 0.05). However, minocycline intervention significantly decreased the latency of radiation group compared with WBI alone (*P* = 0.026) (Figure 2D). The spatial probe test showed no effect of radiation and minocycline treatment on the percentage of target quadrant exploring time (*P* > 0.05) (Figure 2E). These results suggested that minocycline intervention significantly improved the loss of learning and memory ability caused by WBI, and the protective effect was not due to factors such as motor activity and anxiety status.

### Minocycline did not improve hippocampal neurogenesis deficit 2 months post-irradiation

**Figure 3:**
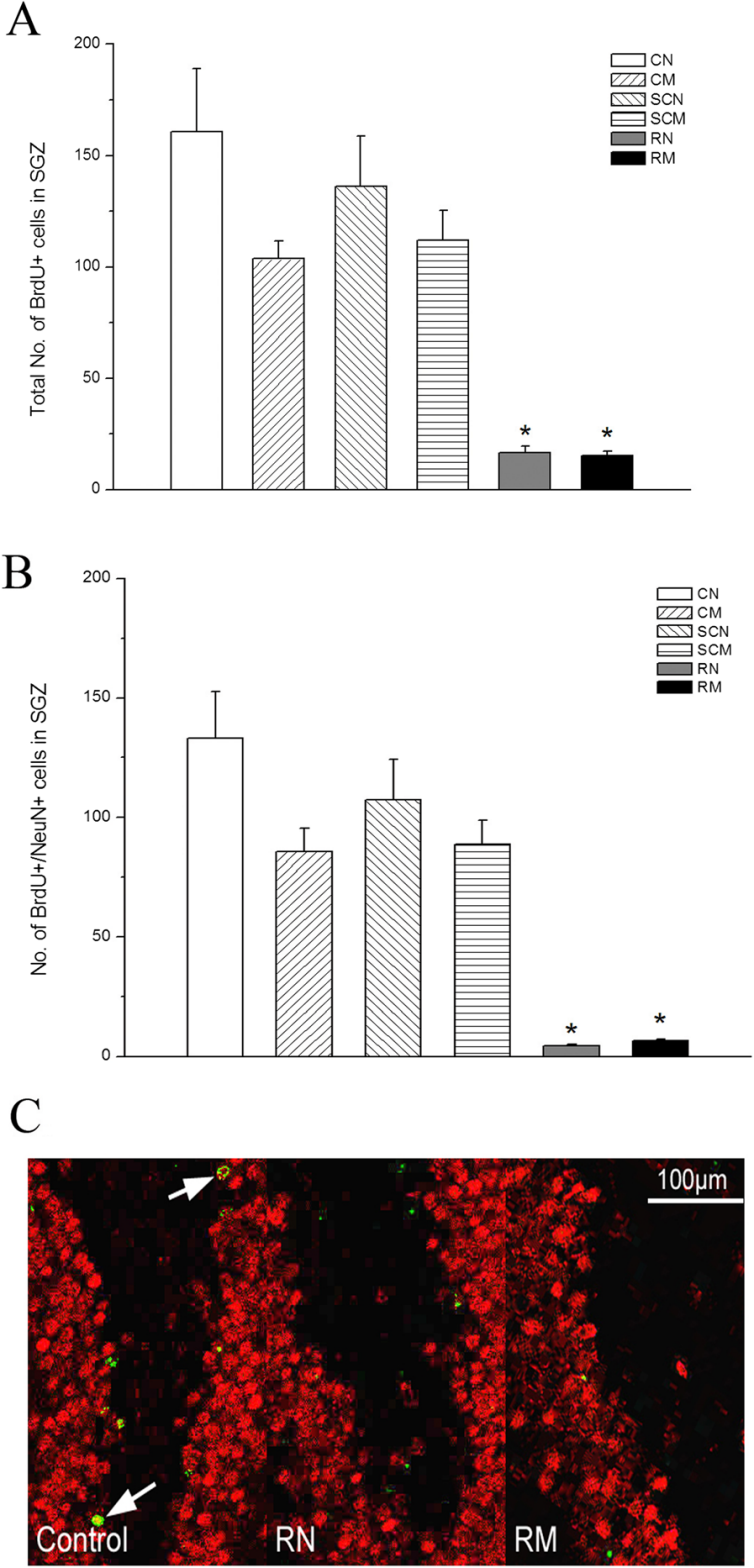
**Hippocampal neurogenesis. (A)** The total number of BrdU^+^ cells in the SGZ two months after WBI. **(B)** Quantification of BrdU^+^/NeuN^+^ cells in the SGZ two months after IR. * *P* < 0.05, compared with the four control groups. **(C)** *In situ* immunohistochemistry images of BrdU^+^ (green) and NeuN^+^ (red) cells in the dentate SGZ two month after WBI. The number of rats: n = 3/group.

The total number of BrdU positive cells in the SGZ was not statistically different among the four control groups e.g. the CN, CM, SCN and the SCM groups (*P* > 0.05) (Figure 3A), suggesting that anesthesia and minocycline did not affect hippocampal neurogenesis. However, two months after receiving a single WBI dose of 20 Gy, the RN group showed a 90% decline in the number of BrdU+ cells (*P <* 0.01). And minocycline intervention did not make any improvement on the decline (*P* > 0.05) (Figure 3A). We also found that irradiation decreased the number of BrdU+/NeuN+ mature neurons by 97% (*P <* 0.01), and minocycline intervention slightly increased that number (4.7 ± 0.3 for the RN group vs 6.7 ± 0.7 for the RM group), but did not reach statistical significance (*P* = 0.055) (Figure 3B, C). The results suggest that minocycline did not have any protective effect on neurogenesis and neuronal differentiation deficit induced by WBI.

### Minocycline decreased radiation-induced apoptosis in neurons shortly after WBI

**Figure 4:**
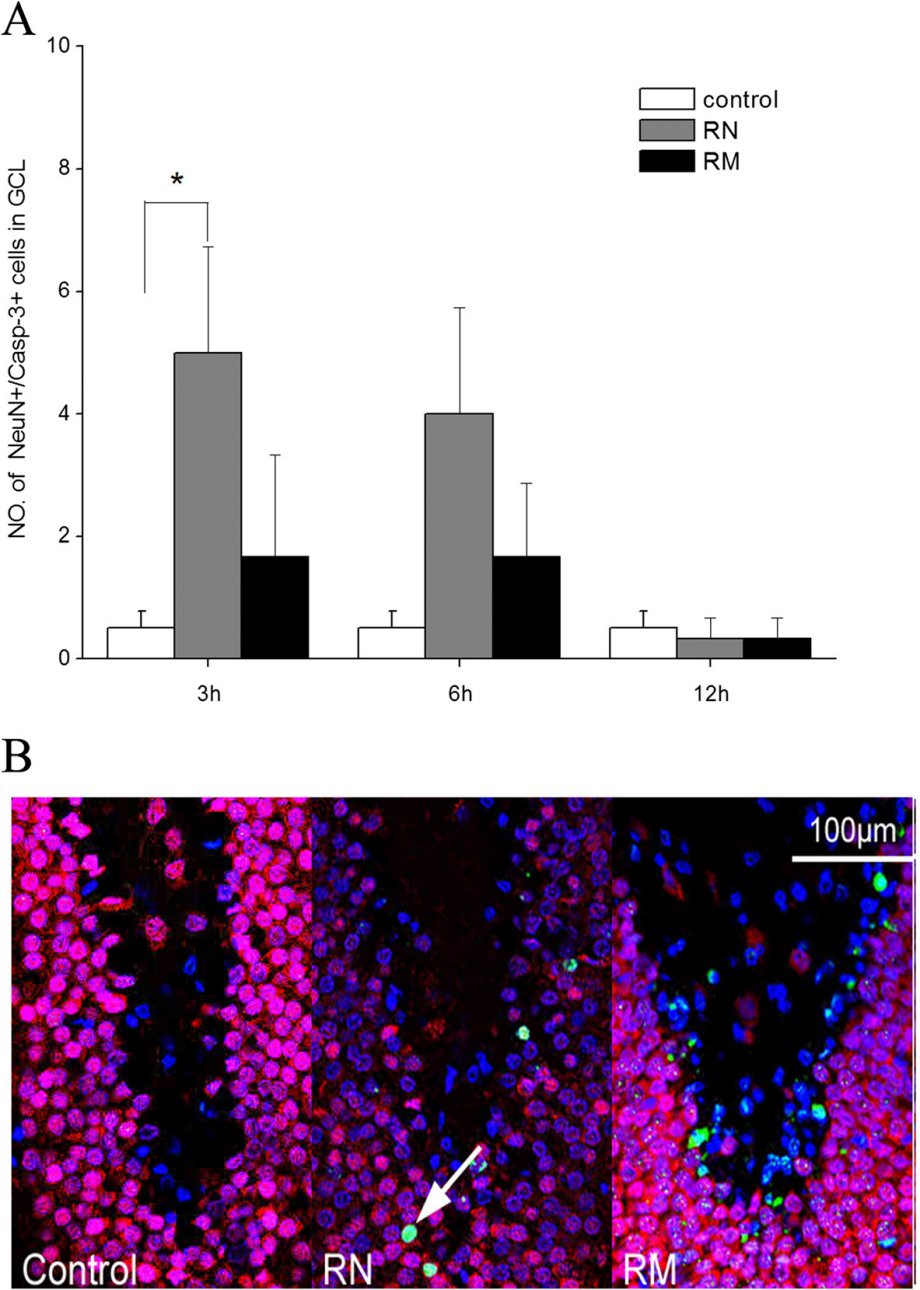
**Radiation-induced apoptosis in the dentate GCL. (A)** The numbers of NeuN+/caspase-3+ cells in the dentate GCL in irradiated rats at different times after irradiation. * *P* < 0.05, compared with the control groups. **(B)** *In situ* immunohisto-chemistry images of the dentate GCL 3 h after WBI. Cell markers are: NeuN (a nuclear antigen in mature neurons, red), caspase-3 (marker for apoptotic cells, green) and DAPI (marker for nuclei, blue). The number of rats: n = 3-4/group.

We found that radiation caused an increase in the number of NeuN+ neurons with activated caspase-3, an established apoptosis marker, in the dentate GCL at 3 and 6 h post-irradiation when compared with the control groups (The four control groups e.g. the CN, CM, SCN and SCM groups showed similar caspase-3 level (data not shown)), with statistical significance only at 3 h (*P = 0.029*) but not 6 h (*P = 0.065*) post WBI. By 12 h after WBI, the numbers of NeuN+ neurons with activated caspase-3 in the RN group were back to the level in the control groups (Figure 4A). Moreover, minocycline intervention decreased the numbers of NeuN+ neurons with activated caspase-3 at 3 and 6 h post-irradiation in the RM group, but did not achieve a significant difference when compared with the RN group (*P* = 0.24, 0.33) (Figure 4A), suggesting that minocycline did not have a strong protective effect on radiation-induced apoptosis in NeuN+ mature neurons in the dentate GCL.

**Figure 5:**
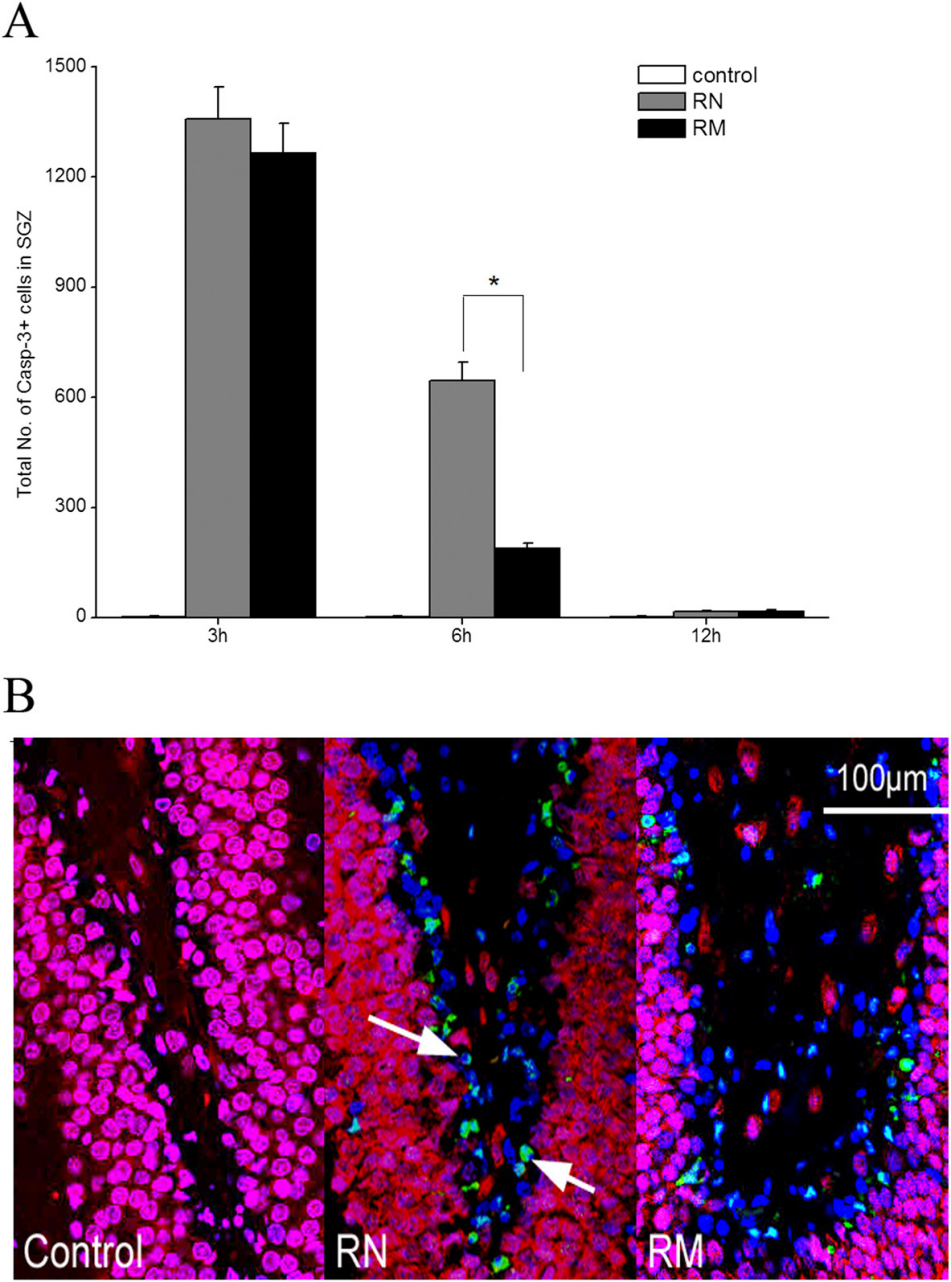
**Radiation-induced apoptosis in the dentate SGZ. (A)** The total numbers of caspase-3+ cells in the dentate SGZ in irradiated rats at different times after irradiation. * *P* < 0.05, compared with the RN group. **(B)** *In situ* immunohistochemistry images of the dentate SGZ 6 h after WBI. Cell markers are: NeuN (red), caspase-3 (green) and DAPI (blue). The number of rats: n = 3-4/group.

In contrast to fewer apoptotic neurons in the dentate GCL post WBI, radiation resulted in a significant increase in apoptosis in the dentate SGZ at 3 and 6 h post-irradiation in the RN group compared with the control groups (*P* < 0.001) (Figure 5A). Similar to the dentate GCL, the four control groups e.g. the CN, CM, SCN and SCM groups showed similar caspase-3 level in the dentate SGZ (data not shown). Minocycline intervention reduced the apoptosis level in the dentate SGZ by 71% at 6 h post-irradiation in the RM group (*P* < 0.001), but did not significantly inhibited radiation-induced apoptosis at 3 h post-irradiation (*P* = 0.48, RM group *vs* RN group) (Figure 5A). These results suggested that minocycline had protective effects on the neurons in the SGZ from radiation-induced apoptosis.

**Figure 6:**
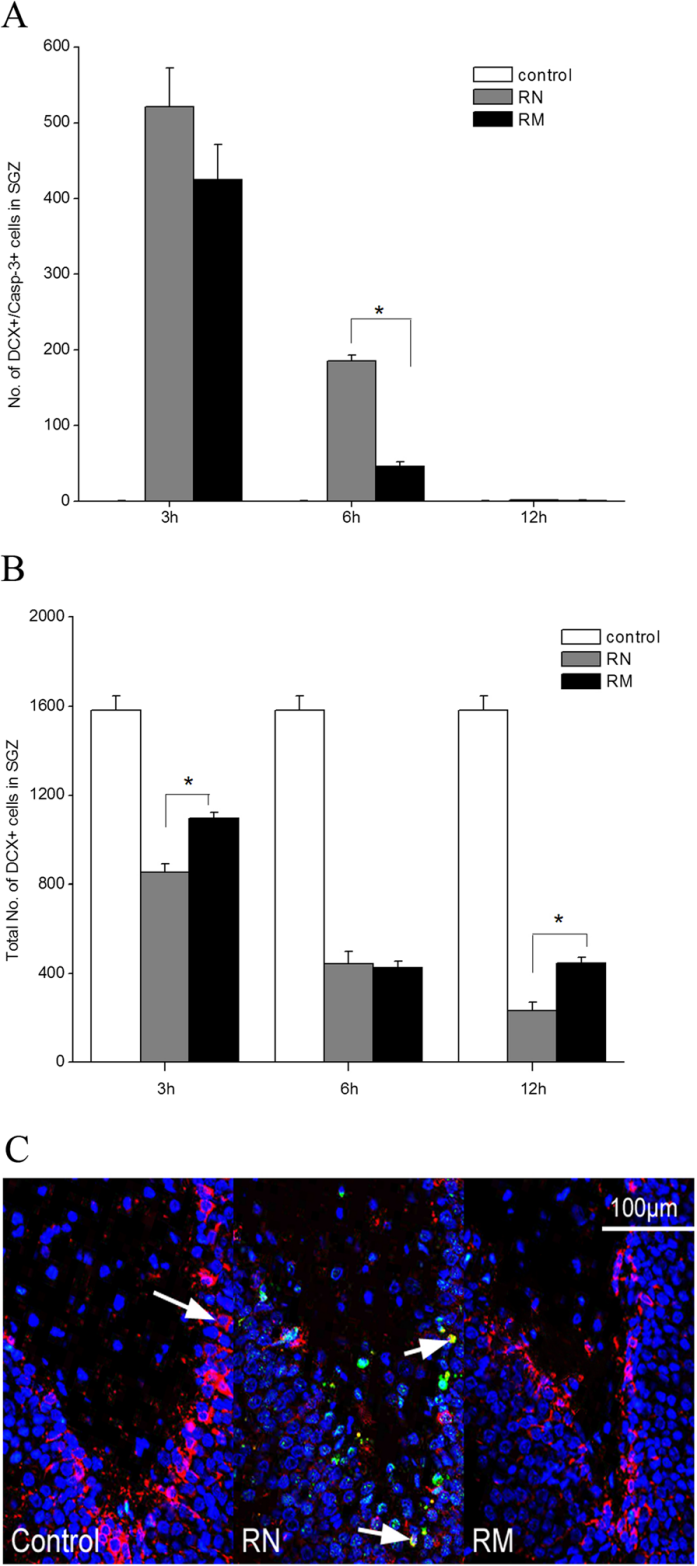
**Minocycline inhibited radiation-induced apoptosis in newborn neurons and decreased the depletion of total number of newborn neurons. (A)** The numbers of DCX+/caspase-3+ cells in the dentate SGZ in irradiated rats at different times after irradiation. * *P* < 0.05, RN vs RM. **(B)** Quantification of the total numbers of DCX+ cells in the SGZ in irradiated rats at different times after irradiation. * *P* < 0.05, RN vs RM. **(C)** *In situ* immunohistochemistry images of the dentate SGZ 6 h after WBI. Cell markers are: DCX (a nuclear antigen in new neurons, red), caspase-3 (green) and DAPI (blue). The number of rats: n = 3-4/group.

To determine whether the observed protective effects of minocycline intervention on the neurons in the SGZ was ascribed to its protective effects on the newborn neurons, a double staining of both DCX (an immature neuron marker) and activated caspase-3 was performed. As shown in Figure 6, the apoptotic DCX+ neurons in the SGZ occurred rarely in the control groups (Figure 6A, C). There was no difference among the four control groups (data not shown). However, WBI induced a significant increase in apoptosis of DCX+ neurons in the SGZ, and the apoptosis level appeared to peak (521 ± 51.1 caspase-3+ cells) at 3 h, then went back to the control level at 12 h post-irradiation (Figure 6A). Minocycline appeared to slightly decrease the apoptosis level at 3 h after irradiation (*P* = 0.24), but significantly reduced the apoptosis level by 75% at 6 h post-irradiation when compared with the RN group (*P* < 0.001) (Figure 6A). The results regarding the effect of minocycline on radiation-induced apoptosis in DCX+ neurons in the SGZ showed similar pattern to its effect on apoptosis in the SGZ, suggesting that the protective effect of minocycline on DCX+ neuron from radiation-induced apoptosis contributed to its protective effect on the SGZ.

DCX+ neurons existed in large numbers in the SGZ, averaging 1583 ± 63 DCX+ neurons in sham-irradiated animals. Irradiation significantly reduced the number of DCX+ neurons in the SGZ by 47%, 72% and 85% for 3, 6 and 12 h post-IR, respectively (*P* < 0.001), and minocycline treatment caused a recovery in the number of DCX-positive cells by 28.4% (*P* = 0.007) at 3 h after irradiation (Figure 6B). The recovery was not observed at 6 h post-irradiation. But by 12 h after radiation, the number of DCX+ neurons in the SGZ in the RM group was 91% greater than that in the RN group (*P* = 0.009) (Figure 6B), suggesting that minocycline intervention could facilitate preservation of DCX+ neurons in the SGZ post-irradiation.

## Discussion

Cranial radiation therapy often causes neurological side-effects including cognitive impairment. Our study has demonstrated for the first time that minocycline, a clinical available antibiotic, can significantly improve the learning and memory loss in rats caused by WBI. Further studies show that minocycline intervention does not have any protective effects on neurogenesis deficit 2 months post-irradiation. However, we found that minocycline can protect the newborn and immature neurons in the dentate SGZ from radiation-induced apoptosis, thus resulting in less newborn neuron depletion shortly after WBI.

The MWM has been used to reveal a severe spatial navigation deficit in the adult rats receiving a single high dose of X-rays (8-9 Gy) shortly after birth [24]. Using the same assay, our previous study has also showed a significant cognition decline in rats exposed to X-irradiation at one-month old [21]. In the present study, using MWM we found that minocycline intervention significantly attenuated cognitive decline in irradiated rats (Figure 2D). Since the learning and memory performance of rats could be affected by their anxiety and motor activity, we also measured their levels of anxiety and swimming speeds, and found that both irradiation and minocycline did not affect them. Thus we could rule out the contribution of anxiety and motor activity to WBI-induced cognitive decline and the protective effect of minocycline in rats.

The mechanisms underlying the decline of cognitive function is unclear, although accumulated evidence suggests that reduced hippocampal neurogenesis adversely affects memory formation [25-29]. Consistent with previous report [11,12], our results demonstrated that radiation-induced learning and memory deficit was accompanied with significant decline of neurogenesis. However, no protective effects of minocycline on neurogenesis was observed in spite of its recovery effect on the learning and memory performance in irradiated rats. The effect of minocycline on neurogenesis seems somewhat controversial. The study from Kohman et al. [30] showed that minocycline may recover some aspects of cognitive decline associated with aging, but the effect appears to be unrelated to adult hippocampal neurogenesis. Ng et al. found that despite the attenuation of activated microglia, minocycline do not support neurogenesis in the hippocampus [31]. However, Mattei et al. recently reported that minocycline rescues decrease in neurogenesis in an animal model of schizophrenia [32].

Moreover, we found that WBI-induced cognitive impairment was accompanied with severe neuron apoptosis, especially apoptosis in the newborn and immature neurons in the dentate SGZ, which agrees with the previous studies [8,9]. Minocycline did not have strong protective effects on radiation-induced apoptosis in mature neurons in the dentate GCL (Figure 4A). In contrast, minocycline protected the newborn and immature neurons in the dentate SGZ from radiation-induced apoptosis (Figures 5A and 6A), thereby resulting in less newborn neuron depletion 12 h after radiation (Figure 6B). This is similar to the previous report that pretreatment with minocycline mitigated isoflurane-induced cognitive deficits and suppressed the isoflurane-induced caspase-3 activation and apoptosis in the hippocampus 4 h after isoflurane exposure [33]. The neuroprotective properties of minocycline have been suggested to be due to its direct antioxidant activity, which is as good as Vitamin E [19]. It has been shown that ionizing radiation can induce caspase-3-dependent apoptosis through generation of reactive oxygen species (ROS) in Neural stem cells [34]. Thus an antioxidant like minocycline could inhibit caspase-3-dependent apoptosis by scavenging ROS. Although Mizumatus et al. showed that acute dose-related changes in SGZ precursor cells qualitatively correlate with later decreases in new neuron production after radiation, and suggested that precursor cell radiation response may play a contributory if not causative role in radiation-induced cognitive impairment [11], linking radiation-induced acute damage in hippocampus at early times to the progressive cognitive impairment at late times after radiation is still difficult. Therefore, despite our results, at this time we are unlikely to be able to draw the conclusion that the recovery effect of minocycline on the decline of cognitive function of rats 2 months post-irradiation was due to its protective effect on neurons from radiation-induced apoptosis shortly after WBI.

It was suggested that the alleviating effect of minocycline on long-term spatial memory impairment in aged mice was associated with the inhibition of astrocytic activation [35]. In addition, minocycline was found to reduce astrocytic reactivation and neuroinflammation in the hippocampus of a vascular cognitive impairment rat model [36]. Therefore, whether the mechanisms underlying the protective effects of minocycline on radiation-induced cognitive impairment involves inhibition of astrocytic activation and neuroinflammation needs to be elucidated in the further studies.

## Conclusions

In summary, we have found minocycline, a clinical available antibiotic, does not affect normal growth, and significantly attenuates irradiation-induced cognitive impairment and protects newborn neurons from radiation-induced apoptosis, leading to less new neuron loss. The results indicate a potential clinical implication of minocycline as an effective adjunct in radiotherapy for brain tumor patients.
